# Stroke-like Symptoms as Presenting Signs of Varicella Zoster Meningitis in an Immunocompetent Adult

**DOI:** 10.7759/cureus.22062

**Published:** 2022-02-09

**Authors:** Eric Landa, Frederick Campos, Saad Javaid, Erika Vigandt, Jung Won

**Affiliations:** 1 Internal Medicine, Unity Health, Searcy, USA; 2 Internal Medicine, Wyckoff Heights Medical Center, Brooklyn, USA; 3 Medicine, Nishtar Hospital, Nishtar Medical University, Multan, PAK; 4 Internal Medicine, The Brooklyn Hospital Center, Brooklyn, USA

**Keywords:** stroke-like symptoms, vzv meningitis, aseptic meningitis, viral meningitis, varicella-zoster virus

## Abstract

Varicella zoster is one of the common causes of aseptic meningitis, typically seen in immunosuppressed individuals and rarely in the immunocompetent. The varicella zoster virus (VZV) infection is normally associated with a dermatomal rash in the abdomen with spread to the back. The small prevalence of VZV in immunocompetent individuals may be due to lack of recognition; thus, it is always important to keep it in mind when meningitis is in the differential. Here, we present a case of varicella zoster meningitis in an immunocompetent adult presenting with slurred speech, dizziness, and episodes of confusion.

## Introduction

Varicella zoster virus (VZV) belongs to the herpesvirus family and presents in a variety of clinical manifestations. It first appears as chickenpox, normally in children, after which it becomes latent in the dorsal root ganglion and can reactivate at any time in life [1]. A stressor, such as immunosuppression, can trigger the reactivation of the virus and manifest as herpes zoster in the way of a herpetic rash in a dermatomal distribution, typically in the abdomen and back. Aseptic meningitis caused by VZV is normally seen in the immunosuppressed but not often in the immunocompetent [1-2]. Varicella zoster meningitis should be suspected in anyone with altered mental status and attributing features of VZV such as a dermatomal rash. We report an atypical case of a 69-year-old woman who presented to the Emergency Department (ED) with stroke-like symptoms and was found to have VZV meningitis.

## Case presentation

A 69-year-old woman with a significant past medical history of diabetes mellitus presented to the ED after waking up in the middle of the night dizzy, which caused her to lose her balance and fall on the floor. She did not lose consciousness. This was witnessed by her mother who stated that since that episode earlier in the day, she had been having episodes of confusion along with new-onset slurred speech. The patient at baseline was alert and oriented to person, place, and time. In the ED, she was complaining of some abdominal pain that spread to her back. On physical examination, her vital signs were significant for a blood pressure of 198/98 mm Hg. Physical examination was significant for an itchy vesiculopapular rash on the right mid abdominal quadrant extending to the back. Given her presentation, one of the top differentials was stroke. She was given Labetalol 30mg intravenously for blood pressure control with permissive hypertension and was started on Valacyclovir for shingles. Labs were significant for a normal white blood cell (WBC) count of 7.51 K/UL, electrolytes were within the normal range, liver function tests were within the normal limit, urine toxicology was unremarkable, and COVID test was negative. A head CT without contrast was negative for any bleeding. A CT angiogram of the head and neck was unremarkable for any significant stenosis of the carotid arteries, and a CT perfusion study was also negative. A follow-up magnetic resonance imaging (MRI) of the brain without contrast demonstrated no acute infarct or acute intracranial findings. After all these tests had come back negative and there was no explanation for the episodes of confusion, meningitis came into the differential and she was started on vancomycin, ceftriaxone, and acyclovir. A lumbar puncture was performed, and serology was sent (Table 1). Cerebrospinal fluid (CSF) culture came back positive for VZV and no other organisms. An HIV or hepatitis panel was not obtained, and therefore immunosuppression could not be completely ruled out; however, there was no suspicion for such infections. Her blood glucose levels during her hospital course were within normal, and therefore a hemoglobin A1C test was felt unnecessary. After completing a course of the appropriate antiviral, her symptoms resolved and she was discharged home.

**Table 1:** CSF analysis demonstrating a VZV infection CSF, cerebrospinal fluid; PCR, polymerase chain reaction; RBC, red blood cell; VZV, varicella zoster virus; WBC, white blood cell

CSF analysis	Results	Normal range
CSF color	Colorless	Not applicable
CSF appearance	Clear	Not applicable
CSF RBC	0	0
CSF WBC	35 CUMM	0-10 CUMM
CSF lymphocytes	100%	40-80%
CSF glucose	56 mg/dL	60-80 mg/dL
CSF protein	62 mg/dL	15-45 mg/dL
CSF PCR	VZV detected	Not applicable

## Discussion

VZV is a member of the herpes virus family and is a common cause of viral infections in the USA [1-2]. The virus spreads through air droplets and affects the nasopharyngeal lymphoid tissue in the initial phase. The infected T cells first replicate in the local lymph nodes and then spread throughout the body, including the epidermis, causing the primary infection (chickenpox) [1-2]. VZV downregulates the major histocompatibility complex (MHC) and inhibits interferon response genes. The prolonged incubation period before the onset of skin lesions is indicative of the time taken by the virus to overcome the local immune-mediated defence [3-5]. After the development of the rash, the virus travels retrograde through sensory nerve endings and becomes latent in the regional ganglia.

The reactivation of the virus is usually associated with abnormal skin sensations of burning, tingling, pain, and itching. It can involve multiple neurons, and thoracic and lumbar neurons are typically affected [2]. The rash usually starts as erythematous papules and progresses to grouped vesicles or bullae as the predominant manifestation [2]. It can also become hemorrhagic in immunocompromised and advanced age groups. The most common complication of VZV infection is post-herpetic neuralgia. Other complications include superimposed bacterial infections, motor neuropathy, ocular involvement, and meningitis. Aseptic meningitis is an infrequent complication, and only 0.5% of patients affected with VZV develop meningitis [6-7]. Enteroviruses remain the most common etiology of aseptic meningitis. A review of 859 patients with herpes zoster infection showed that 100 patients developed complications within 60 days of infection, and among those, older age was associated with a higher risk of infections [7].

The pathophysiology of VZV meningitis is still unclear, and it is proposed that the virus can spread through the afferent nerves to the central nervous system and the meninges. Hematogenous spread to the nerves and blood vessels of the central nervous system was the other possible mechanism taken into consideration [8].

The most common symptoms associated with VZV meningitis include headache, fever, or neck stiffness, which were absent in our case; somewhat atypical presentation with dizziness, loss of balance, and diarrhea along with abdominal pain was noted [9]. When the presentation is atypical and nonspecific, as is the case we discussed, the clinical judgment could be misleading. In these circumstances, laboratory confirmation is necessary. CSF polymerase chain reaction (PCR) is the most sensitive test and is widely available now. It is positive in almost 100% of cases [10-11].

CSF PCR shows elevated WBCs and proteins with hypoglycorrhachia. A retrospective study was conducted on 620 patients with meningitis. The study showed that of all these patients, 19% had hypoglycorrhachia on CSF PCR, of which 15% was caused by viruses [11]. Even though the most common cause of hypoglycorrhachia is bacterial in origin, it correlates with the level of hypoglycorrhachia. Patients with CSF glucose of <30mg/dL have a high likelihood of bacterial etiology as opposed to low levels of hypoglycorrhachia in VZV [12-15]. Patients with hypoglycorrhachia in VZV meningitis usually have a history of IV drug abuse. They are more likely to be immunocompromised, unlike the case we presented here with an immunocompetent state and no IV substance abuse. If a PCR test is not available, alternatives include direct fluorescent antibody (DFA) testing on scrapings from skin lesions or viral CSF culture. DFA and viral culture have significantly low sensitivity compared to the CSF PCR (55% and 33%, respectively, vs. 100%), and CSF PCR remains the standard gold test to diagnose VZV meningitis [2,16-18].

There are numerous recommendations regarding the treatment of VZV meningitis. IV acyclovir for two weeks has shown promising results in the resolution of symptoms. The duration may be extended in case of failure to initial treatment. Oral acyclovir has a low bioavailability. Valacyclovir has proven to provide necessary cerebrospinal fluid levels of acyclovir, an acceptable oral option, especially in the outpatient setting [18].

Our patient was effectively treated with a total course of two weeks and was discharged with the resolution of symptoms, and no complications were noted on follow-up as an outpatient.

## Conclusions

Although aseptic meningitis is a common cause of meningitis, VZV is not commonly encountered too often, but when it does present itself, it normally affects those who are immunocompromised. Its presentation, including a rash in the body, is a big clue that should not be overlooked as it can point to the underlying culprit. Lumbar puncture with CSF analysis can help point toward a viral versus bacterial etiology, but PCR is the gold standard for diagnosing VZV. We have presented a rare case VZV meningitis in an immunocompetent individual presenting with atypical symptoms. We hope that healthcare providers will take this case as a reminder that sometimes aseptic meningitis can present atypically and should be kept as a differential in patients presenting with neurological deficits.
